# Skin microbiome modulation induced by probiotic solutions

**DOI:** 10.1186/s40168-019-0709-3

**Published:** 2019-06-24

**Authors:** Bernhard Paetzold, Jesse R. Willis, João Pereira de Lima, Nastassia Knödlseder, Holger Brüggemann, Sven R. Quist, Toni Gabaldón, Marc Güell

**Affiliations:** 1 S-Biomedic, Turnhoutseweg 30, 2340 Beerse, Belgium; 2 Bioinformatics and Genomics Program, Centre for Genomic Regulation (CRG), C. Dr. Aiguader 88, 08003 Barcelona, Spain; 3 Department of Experimental and Health Sciences, Universitat Pompeu Fabra (UPF), C. Dr. Aiguader 88, 08003 Barcelona, Spain; 4 Department of Dermatology, Otto-von-Guericke-Universität Magdeburg, Leipziger Str. 44, 39112 Magdeburg, Saxony-Anhalt Germany; 5 Institució Catalana de Recerca i Estudis Avançats (ICREA), Pg. Lluís Companys 23, 08010 Barcelona, Spain; 6 Department of Biomedicine, Aarhus University, Bartholins Allé 6, 8000 Aarrhus, Denmark

**Keywords:** Skin microbiome, *Cutibacterium acnes*, Microbiome transplantation

## Abstract

**Background:**

The skin is colonized by a large number of microorganisms, most of which are beneficial or harmless. However, disease states of skin have specific microbiome compositions that are different from those of healthy skin. Gut microbiome modulation through fecal transplant has been proven as a valid therapeutic strategy in diseases such as *Clostridium difficile* infections. Therefore, techniques to modulate the skin microbiome composition may become an interesting therapeutic option in diseases affecting the skin such as psoriasis or acne vulgaris.

**Methods:**

Here, we have used mixtures of different skin microbiome components to alter the composition of recipient skin microbiomes.

**Results:**

We show that after sequential applications of a donor microbiome, the recipient microbiome becomes more similar to the donor. After intervention, an initial week-long phase is characterized by the dominance of donor strains. The level of engraftment depends on the composition of the recipient and donor microbiomes, and the applied bacterial load. We observed higher engraftment using a multi-strain donor solution with recipient skin rich in *Cutibacterium acnes* subtype H1 and *Leifsonia*.

**Conclusions:**

We have demonstrated the use of living bacteria to modulate skin microbiome composition.

**Electronic supplementary material:**

The online version of this article (10.1186/s40168-019-0709-3) contains supplementary material, which is available to authorized users.

## Background

The human body is host to a complex and rich microbial community. The human microbiota mainly resides on the skin, on the oral mucosa, and in the gastrointestinal tracts, and has fundamental roles in health and disease [1]. The development of next generation sequencing (NGS) technologies has allowed for the study of these communities with an unprecedented depth and resolution [2]. The gut microbiome has been investigated extensively [3], with the skin microbiome becoming another focus of research more recently [4–8]. The skin is colonized by a large number of diverse microorganisms, of which most are beneficial or harmless [9]. More specifically, microbes colonize the stratum corneum of the epidermis and skin appendages such as sweat glands and hair follicles. The composition of abundant species is relatively stable over time [10]. However, skin-associated diseases such as acne vulgaris [11], eczema [10, 12–14], psoriasis [15], or dandruff [16, 17] are associated with strong and specific microbiome alterations. For instance, the appearance of acne vulgaris has been linked to dysbiosis in the skin microbiome [11, 18]. This distortion is probably caused by a specific subset of the skin bacterium *Cutibacterium acnes* [11, 18–20]. Different strains of this bacterium have different degrees of association with acne. For instance, the presence of strains carrying locus 2, a 20 kb genomic island, is highly associated with the disease [20]*.* Conversely, different *C. acnes* strains have been associated with multiple positive properties [21]. The targeted manipulation of the human microbiome may become a potential therapeutic strategy for the treatment and study of diseases. The most prominent example of this therapeutic principle is the treatment of the antibiotic-resistant bacteria *Clostridium difficile* within the gut microbiome with the help of fecal transplantation [22]. Following this successful treatment, a number of projects are developing microbiome-based treatments for gut diseases [23]. Similarly, manipulation of the skin microbiome entails the promise of novel therapeutic approaches for skin diseases [24].

We are particularly interested in *C. acnes* and its strain diversity, as this bacterium represents a major part of the human skin microbiome, and certain strains are associated with acne vulgaris [11, 18, 25]. Therefore, we developed and tested an approach to modulate the subpopulation of this species at the strain level.

## Results

In this work, we aimed to demonstrate that the human skin microbiome composition can be modulated through approaches similar to those used in fecal transplantation of the gut microbiome. For this, we prepared probiotic solutions from donor microbiomes and applied them onto healthy volunteers, whose skin microbiome was monitored during and after the treatment. Two of these solutions comprise complete microbiome isolations from two donors (CM samples: CM1 and CM2; Additional file 1: Table S1), and three others are composed of defined sets of *C. acnes* strains isolated from donors (PA solutions: H1, H1+A1, and H1+D1+A1; Additional file 1: Table S1). The label “PA” stems from *Propionibacterium acnes*, the original name of the species before it was reclassified as *Cutibacterium acnes* [26].

**Fig. 1:**
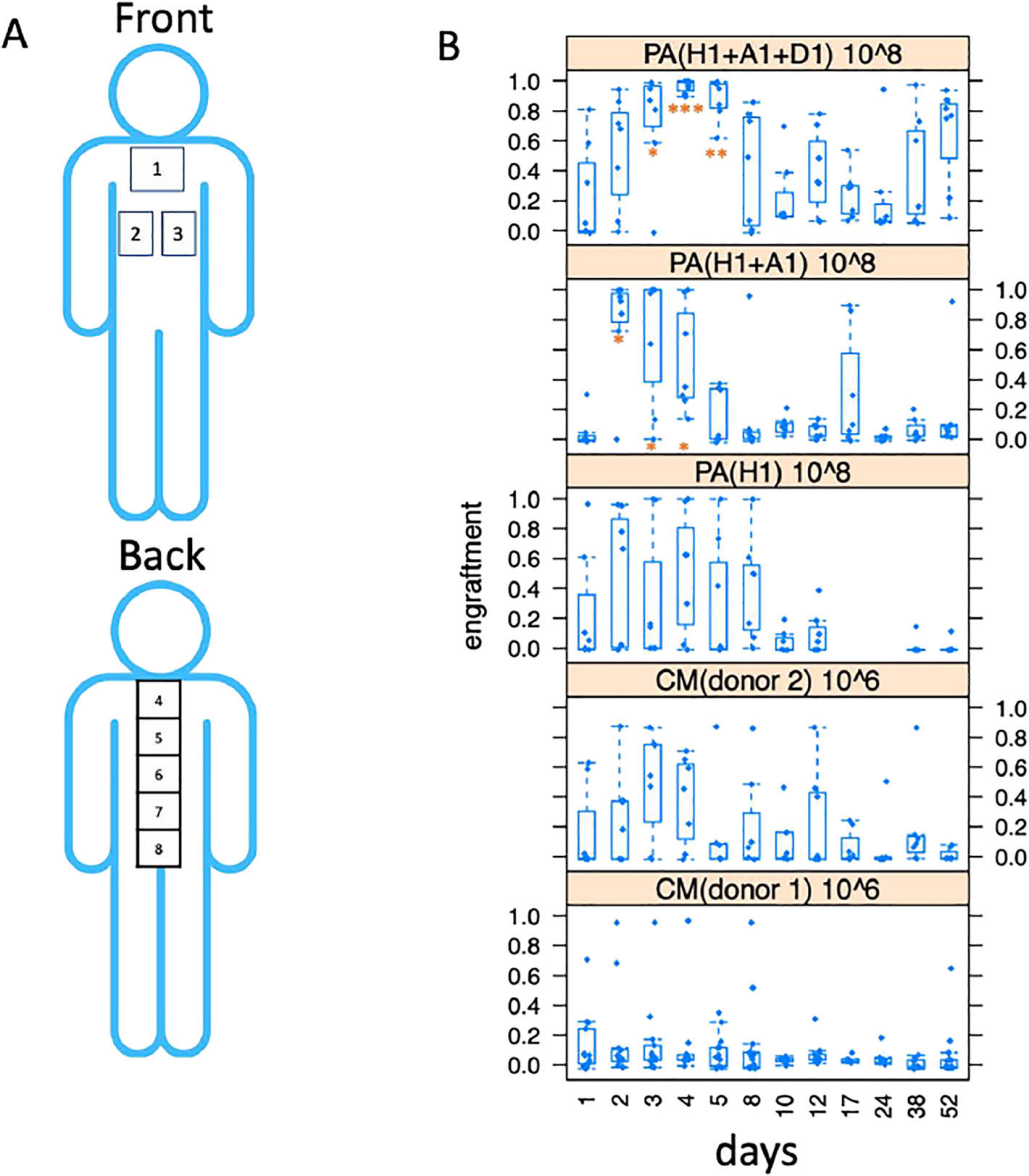
Skin microbiome composition dynamics after donor transplantation. **a** Skin surface areas of study. The squares indicate the areas of application. **b** Engraftment level of different probiotic solutions at different days of application (see Additional file 1: Figure S3 for individual patient information). Point 1 is measured before any probiotic application is carried out. *p value < 0.05. **p value < 0.01. ***p value < 0.005

These solutions were applied on 18 healthy subjects with ages ranging from 22 to 42. Eight different skin areas were defined for application, whereof three were on the chest and five were located along the spine (Fig. 1a). These areas were chosen due to their typically high abundance of sebaceous glands. To get an understanding about the dose response of applied bacterial strains, three different concentrations were chosen (10^4^, 10^6^, and 10^8^ CFU/mL) and applied on the different areas. One area (area 4) was used as a negative control (i.e., no application). To better understand synergistic effects, different strain combinations were used. One mixture contained only strain H1 (H1), a second was spiked with small amounts of A1 (H1+A1), and a third consisting of nearly equal amounts of H1 and D1 and small amounts of A1 (H1+D1+A1). H1 is a type IB strain; A1 and D1 are type IA strains (Additional file 1: Table S2). To circumvent biases on each subject area, a different concentration was applied and rotated along the different individuals. We rotated site application for a given solution to prevent potential specific site biases. Initially assigned treatments were maintained for the rest of the study. All test areas except area 4 (control) were sterilized before application. Probiotic solutions were applied every day during days 1, 2, and 3. Skin microbiome samples were taken with commercial skin stripping method (3S-Biokit, C+K electronic) based on fast hardening cyanoacrylate glue at 16 time points (0, 1, 2, 3, 4, 5, 8, 10, 12, 17, 24, 38, 52 days) to monitor microbiome dynamics (Fig. 1b). DNA was recovered from the strip with a high-temperature extraction solution (see the “Methods” section). We included sampling at day 0 before any probiotic solution was applied. Genomic DNA was extracted and sequenced by NGS-based genotyping.16S rRNA gene profiling was used to assess microbiome composition at the genus level. SLST profiling [27] was used to identify relative proportions of different *C. acnes* strains. Barcoded libraries were constructed and sequenced by an Illumina Miseq machine (Illumina, USA). The obtained data was quality filtered, mapped, and clustered (see the “Methods” section).

**Fig. 2:**
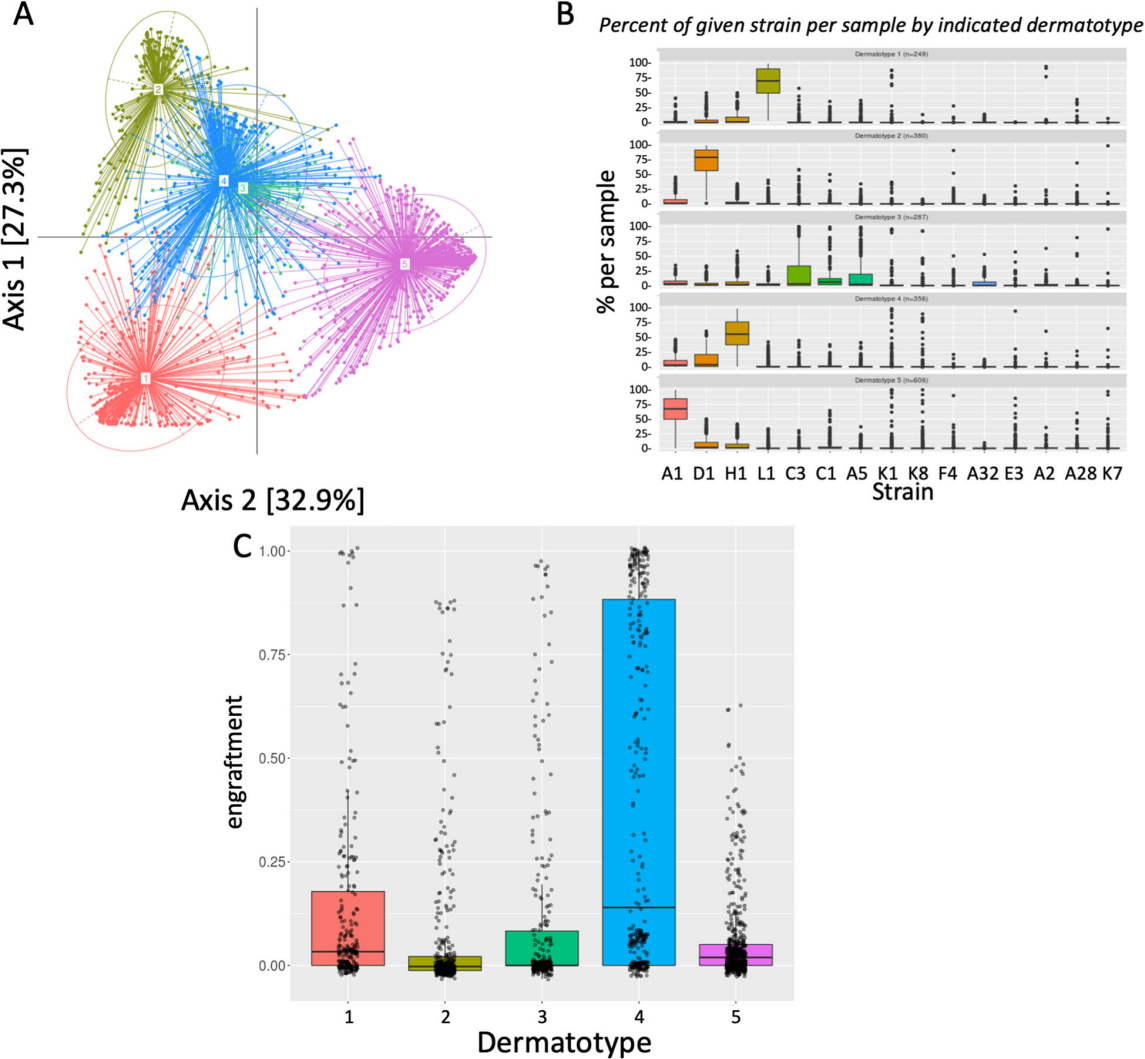
*C. acnes* population dynamics using SLST typing. **a** PCA representation of the different dermatotypes (based on SLST typing). Dermatotypes 3 and 4 appear to have overlap, but separate well in the 3rd axis (19.6% of variance—not shown here). **b** Composition of the dermatotypes (based on SLST typing, see Additional file 1: Table S2 for ribotype equivalences). **c** Average engraftment of different dermatotypes (based on SLST typing)

After the SLST profiling, we performed a partitioning around medoids (PAM) cluster analysis of the samples from all recipients at each time point based on Jensen-Shannon Divergence (JSD) distance, and used a Calinski-Harabasz (CH) index as well as average silhouette width to determine the optimal number of clusters [28, 29] (Fig. 2a). Based on this analysis, we could identify five main clusters of *C.* acnes profiles on the skin. We decided to name these five clusters dermatotypes 1, 2, 3, 4, and 5, analogous to the term enterotype defined for the gut microbiome [29] or stomatotype for the oral microbiome [30]. This classification is helpful to study groups of subjects having similar microbiomes, and it facilitates finding functional associations of certain microbiome signatures. These dermatotypes do not, however, describe discrete clusters with fixed abundances of particular strains, but rather show gradients of variability in these abundances between samples. The skin microbiomes of dermatotype 1 are driven by *C. acnes* L1, dermatotype 2 by *C. acnes* D1, dermatotype 3 by C3 and A5, dermatotype 4 by D1 and H1, and dermatotype 5 by *C. acnes* A1 (Fig. 2b). Second, we observed a quantitative and qualitative increase in similarity between donor and recipient microbiomes after only 3 days of application. For each solution, we assessed engraftment levels (Figs. 1b and 2c; Additional file 1: Figure S1) and the change of the composition of the *C. acnes* subpopulation before the treatment at three predetermined concentrations (10^4^, 10^6^, and 10^8^ CFU/mL; see Additional file 1: Table S1). Engraftment is measured as the distance between the microbiome composition of the tested sample and the applied solution (see the “Methods” section).

Despite the fact that subjects were allowed to shower and practice sports, engraftment is detected for multiple samples well beyond the application days. Some of the applied mixtures engraft better. PA mixtures engraft better than CM at any concentration, and the highest concentration (PA8 or 10^8^ CFU/mL) has significantly higher engraftment values (Additional file 1: Figures S1 and S2). The values show that engraftment is greater with the H1+A1+D1 solution, followed by H1+A1 and (H1), in this order (Additional file 1: Figure S1). Expectedly, higher concentrations show greater engraftments (Additional file 1: Figure S2). PA8 containing H1, A1, and D1 engrafts significantly better than all the other groups. Both of the CM samples engraft less than PA. CM from donor 1 engrafts less than CM donor 2 (see donor compositions in Additional file 1: Figure S3). Interestingly, donor 2 has a 1.8-fold greater ratio of *C. acnes* over *Staphylococcus* species (*Staphylococcus aureus* and *Staphylococcus epidermidis*) than donor 1.

Not all subjects responded equally to the applied samples, indicating significant variability among recipient areas that sometimes relate to defined *C. acnes*-based dermatotypes. Indeed, we measure some variability on subject-to-subject results (Additional file 1: Figure S4); oscillations observed for a single patient in particular days are not considered significant. For instance, dermatotype 4 shows higher engraftment than others (Fig. 2c, Tukey’s test). Interestingly, this dermatotype is dominated by H1 and comprises notable levels of D1 and A1 (Fig. 2c). We did not observe an association between Shannon diversity of individual subjects and engraftment levels (Additional file 1: Figure S5).

**Fig. 3:**
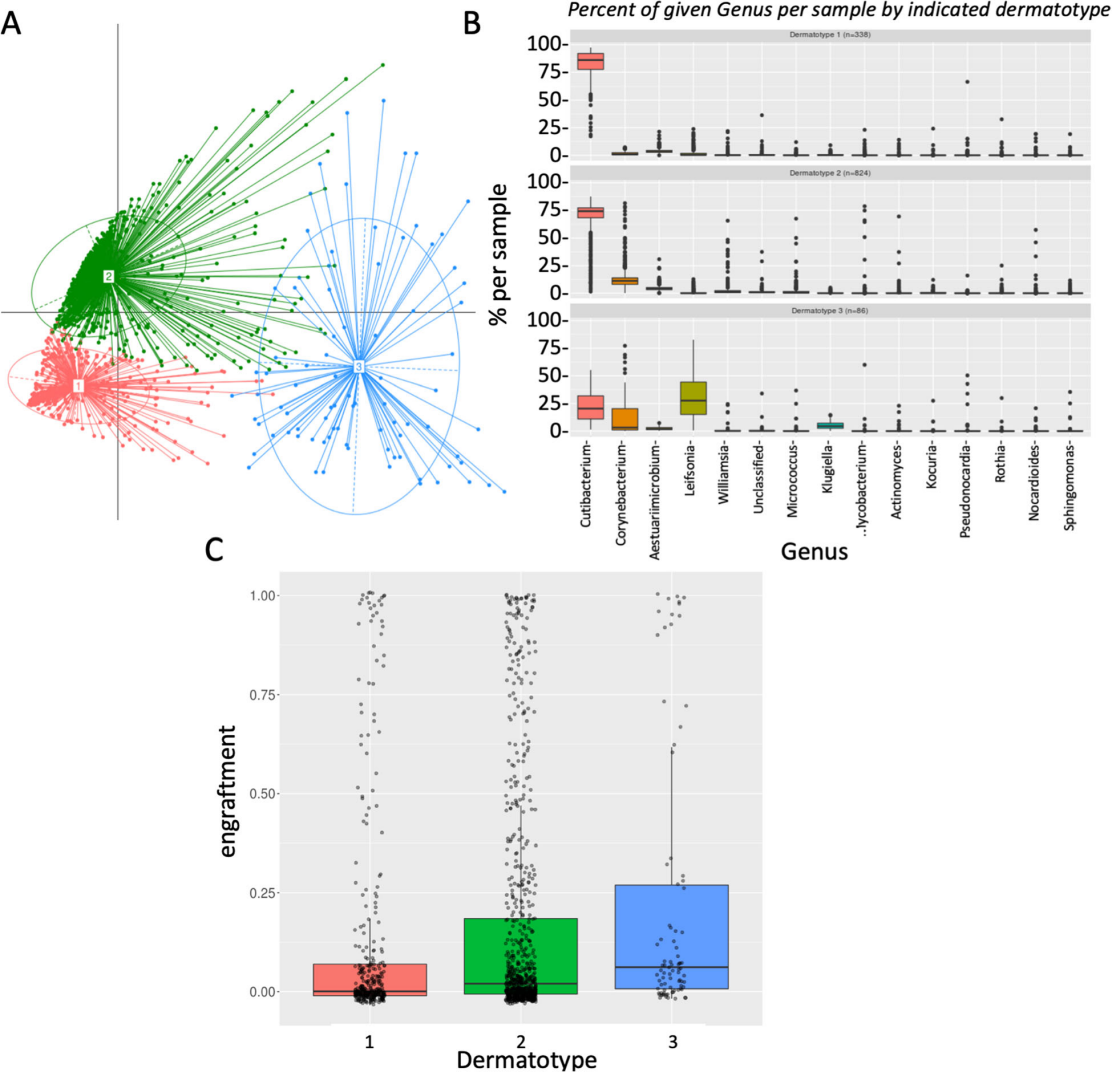
Complete microbiome dynamics at the 16S level. **a** PCA representation of the different dermatotypes (based on 16S typing). **b** Composition of the dermatotypes (based on 16S typing). **c** Average engraftment of different dermatotypes (based on 16S typing)

We also classified patients according to the different 16S-based dermatotypes. In this case, we observe 3 different types: type one dominated by *Cutibacterium*, type two dominated with *Cutibacterium* and some *Corynebacterium*, and a more widespread type 3, with *Leifsonia* being more abundant (Fig. 3a, b). Interestingly, we observe important anticorrelation between *Cutibacterium* and *Corynebacterium*. *Leifsonia* seems to show more co-occurrence with *Corynebacterium* (Additional file 1: Figure S6). Patients with type 3 show a significantly higher engraftment (Fig. 3c). We hypothesize that patients of type 3 are not fully colonized with *Cutibacterium*, and therefore, it is easier to establish a new population.

## Discussion

We have demonstrated that the composition of the human skin can be modulated by applying *C. acnes* strain H1 with positive features isolated from healthy individuals [31–33]. Combining H1 (type IB) with other strains such as D1 (type IA) and A1 (type IA) significantly enhances engraftment (Fig. 1b). A1 is the most widespread strain, and D1 has been described as not being associated with acne [11]. All three strains were observed to grow well in vitro during the study. We have combined H1 with two other strains and measured superior engraftment of the complex mixtures.

The dose of applied bacteria plays an important role in the modulation capacity. During the first three days, the abundance of applied bacteria increases each day and then decreases gradually after the termination of application. The applied dose determines the prolongation of the abundance of the applied strain on the tested skin.

This return to the ground state is in accordance with a recent study reporting temporal stability of the skin microbiome [5]. Unfortunately, our data is limited to skin areas rich in sebaceous glands and we do not know whether other areas which are suspected to be more dynamic [6] and involved in other diseases [13] react differently. This is an interesting question as different body sites harbor different bacterial subpopulations [27] and their reaction to external modulation might be different.

Overall, this study shows that a temporary modulation of the *C. acnes* population at the strain level is feasible without a negative reaction of the host. All subjects underwent dermatological inspection, and no adverse effects were detected. This gives researchers a new tool to probe hypotheses based on the association of the *C. acnes* population with skin diseases [15, 17, 34]. We are looking forward to more research involving skin microbiome modulation to shed light on the role of microbiome dysbiosis in disease.

## Conclusions

Microbes are important components of the skin. Recent clinical studies already revealed that application of natural bacteria into the skin can decrease skin pH and improve moisture retention [35]. This method opens the possibility to develop probiotic solutions that help the human skin reverting disease microbiome states to healthy ones. Also, synthetic biology is generating smart microbes with the abilities to detect and treat disease [36]. New methods to replace and modulate our bacterial flora are necessary. We expect that this methodology could be used to study and modify skin microbial components and have broad implications for future therapies and research in skin microbiome and related diseases.

## Methods

### Donor and recipient group definition

Donors were healthy males and females between 22 and 40 years old, male and female, healthy individuals. Healthy individual is understood as a subject without visible skin problems and not immunocompromised. Subjects were evaluated visually by the researcher or dermatologist who ran the study and took samples. Additionally, written consent on the subject health was required before starting the study with each individual subject.

### *C. acnes* strain isolation from the donors (PA mixtures)

A defined mixture of biologically active probiotic bacteria for topical administration was prepared as follows. A sample of skin microbiome was taken from a donor (forehead). The sample was then cultured in the laboratory, and a formulation was prepared.

Methods for analyzing the microbiome included DNA isolation, SLST amplification, and large-scale amplicon sequencing, as well as bioinformatics for the taxonomic assignment and quantification of diversity in microbial communities. Steps included are as follows:

1. Isolation of bacterial strains from a donor. Bacteria were collected using swabs. Swabs were moistened with water.
2. Growth in the laboratory. Bacteria were grown in reinforced clostridium agar (RCM) in anaerobic conditions at 37 °C.
3. Isolation and manipulation of the bacterial strains. The sample was enriched for 20 *Cutibacterium* strains and analyzed for positive genotypes with SLST primers. Strains H1, A1, and D1 were selected.
4. Formulation of a probiotic based on strains H1, A1, and D1. Colonies were picked and grown in liquid RCM medium, and spun down and resuspended in a saline solution with 0.5% peptone.
5. Application of 1mL of the probiotic suspension from step 4 to the recipient. The donor microbiome was applied using swabs, and the area was left to dry.
6. Genotyping of the modified recipient microbiome using an NGS-based genotyping approach discussed below.

### Complete microbiome isolation from the donors (CM mixtures)

A mixture of biologically active probiotic bacteria for topical administration based on complete microbiomes was prepared as follows. A sample of skin microbiome was taken from a donor. The sample was then cultured in the laboratory, and a formulation was prepared. It is important to mention that by growing in vitro the microbiome, donor composition may become biased. Complete microbiome does not refer to comprehensive transfer from donor to acceptor but not-enriched donor sample preparation.

Methods for analyzing the microbiome included DNA isolation, 16S amplification, and large-scale amplicon sequencing, as well as bioinformatics for the taxonomic assignment and quantification of diversity in microbial communities. Steps included are as follows:

1. Isolation of bacterial strains from a donor. Bacteria were collected using swabs. Swabs were moistened with water.
2. Growth in the laboratory. Bacteria were grown in RCM agar in anaerobic conditions at 37 °C.
3. All colonies were collected from plates, grown in liquid RCM medium, and spun down and resuspended in a saline solution with 0.5% peptone.
4. Application of 1 mL of the probiotic from step 3 to the recipient. The donor microbiome was applied using swabs, and the area was left to dry.
5. Genotyping of the modified recipient microbiome using an NGS-based genotyping approach discussed below.

### Skin microbiome donor preparation viability

PA and CM mixtures were grown in RCM as a liquid culture. After 2 days, the culture was spun down and washed first with PBS (phosphate-buffered saline, pH 7.4), and then with water. The pellet was resuspended to a final concentration of PBS and 0.5% peptone. Aliquots were stored either at room temperature or at 4 °C. In both cases, they were protected from sunlight. In regular intervals, about every 3–4 days, a dilution series of each sample was taken and the colony-forming unit (CFU) count was determined. The suspension was vortexed, and a serial dilution was prepared. To determine the CFU count, aliquots of the dilution were added on agar plates which are suitable to grow *C. acnes*. A volume of 10 μl of an appropriate dilution was introduced to RCM plates. The 10 μl were placed as a drop on top of the plate and run down. This method allows the placement of up to 4 drops on the plate. Each sample was determined in 4 technical replicates. After 3–4 days of anaerobic incubation, the colony numbers were counted (manually or using the software OpenCFU) and both the average and the standard deviation were determined. Thereby, a profile of the colony forming units was monitored over time. Combined samples (i.e., H1+D1; H1+D1+A1) were always mixed freshly before deployment https://www.future-science.com/doi/pdf/10.2144/97234bm22 [37].

Additionally, we performed some stability study of the probiotic solutions. Bacteria of the skin microbiome were stabilized in a neutral liquid matrix for several days at room temperature (i.e., saline solution or 0.5% peptone). It was demonstrated that *Cutibacterium* can survive weeks of storage at room temperature. Constant numbers of colony-forming units (CFUs) from a liquid matrix over a week were also recovered. To assess these numbers, methods that determine the CFU of liquids in a medium-throughput fashion were established as described below. It was shown that compositions were stable for at least 1.5 months.

### Donor microbiome solution application to the recipient

Microbiome donor solution was applied once a day for 3 days using swabs onto a delimited area of the chest of the recipient (Fig. 1a). Prior to application, the area was cleaned and disinfected. Sampling for genotyping was carried out before new donor samples were applied.

Treatment areas measure 100 cm^2^ so that sampling can be performed in adjacent sides and prevent potential measurement artifacts associated with using 3S-Biokit repeatedly at the exact same point.

Different areas of sampling defined in the study may have slightly different properties (i.e., variation of pilosebaceous unit) and experience different environmental features (access to wash, clothes contact, etc.). Due to the lack of previous reports of this study, we prioritized perhaps a noisier but less biased sampling scheme. A rotatory scheme may prevent the observation of results that may be very unique to one specific site and provide more generalizable results.

### Engraftment

In this study, we used natural strains that cannot be distinguished between donor and acceptor. We determined the engraftment computing the differences in the proportions of strains present in the acceptor and in the applied solution. We defined engraftment as the distance between the composition of the site and the applied solution. The distance was computed using Pearson’s correlation.

### Strain genotyping

An NGS-based genotyping approach was used for identifying different strains:

1. The microbiome was collected using strips daily. Strip kit 3S-Biokit from C+K electronic was used.
2. The sample was incubated at high temperature to isolate the DNA. The QuickExtract™ kit from Epicentre, Chicago, IL, was used with some modifications. A volume of 80 μL of 0.05 M NaOH was added to the suspension solution. The incubation was conducted for 45 min at 60 °C, followed by a 5-min incubation at 95 °C. After incubation, 920 μL of 1 M Tris-HCl (pH 7.0) was added. A volume of 0.5 μL was used for PCR.
3. PCR was conducted on the sample using 16S primers, and SLST primers characterize the population. Samples were amplified using KAPA polymerase (Initial denaturation for 5 min at 95 °C followed by 35 cycles of 98 °C for 20 s, 62 °C for 25 s, and 72 °C 30 s; and a final elongation for 1 min at 72 °C).

Primers used for 16S amplification were as follows:

**5’-TCGTCGGCAGCGTCAGATGTGTATAAGAGACAG**-*CCTACGGGNGGCWGCAG-3’* and

5’-**GTCTCGTGGGCTCGGAGATGTGTATAAGAGACAG**-*GACTACHVGGGTATCTAATCC-3’*

The Illumina overhang adapter sequence is in bold, and the 16S V3 and V4 region priming sequence described in Klindworth et al. [38] is in italics.

Primers used for SLST amplification were as follows:

**5’-TCGTCGGCAGCGTCAGATGTGTATAAGAGACAG**-*TTGCTCGCAACTGCAAGCA-3’* and

5’-**GTCTCGTGGGCTCGGAGATGTGTATAAGAGACAG**-*CCGGCTGGCAAATGAGGCAT* -3’

The Illumina overhang adapter sequence is in bold, and the sequence used for SLST targeting is in italics. We used a shorter SLST amplicon version which can be fully sequenced by Miseq PE300 Illumina sequencing:

4. Library preparation. The library was constructed using two rounds of PCR. The 10 first round used 16S primers and SLST primers which included sequences compatible with Illumina sequencing. The second round was used to barcode the different samples for sequencing in a single Illumina flow cell.
5. Illumina MiSeq sequencing was conducted. We reserved a depth of ~ 10,000 reads for each sample.
6. Samples were analyzed using a two computational pipeline for 16S and SLST. The 16S pipeline was performed as described in Willis et al. [30]. SLST typing pipeline consisted on quality filtering; SLST amplicon was mapped to SLST database [27] using BWA mapping software; BAM file processing and visualization were conducted with R statistical language.

### Significance of engraftment

We performed Wilcoxon-Mann tests between measured engraftment before treatment (day 1) and after treatment (days 2, 3, 4, 5, 8, 10, 12 ,17, 24, 38, and 72). Multiple testing was adjusted using the BH method.

### Normalization and filtering of 16S and SLST data

The 16S rRNA gene counts and the SLST counts for the samples in this study were stored and analyzed using the R package Phyloseq (version 1.16.2) [36]. The counts were normalized per sample by dividing each value by the sum of all counts for a given sample and multiplying by 100, leaving the relative abundance of each genus/strain within that sample, with all values between 0 and 100.

### Clustering and dermatotype analyses

The Jensen-Shannon divergence (JSD) was used to produce a distance matrix between the genera/strains of all samples and then partitioning around medoids (PAM) clustering to group samples with similar overall abundances. We used the Calinski-Harabasz (CH) index to determine the optimal number of clusters, and we further verified this by calculating the average silhouette width of the samples, which is a measure of the separation of samples within one cluster from those of another cluster. The functions for these calculations come from the R packages cluster (version 2.0.4) [38] and clusterSim (version 0.44-2) [38]. A Principal Coordinate Analysis (PcoA) was used to visualize the clustering of the samples within their respective dermatotypes with the R package ade4 (version 1.7-4) [39].

## Additional files

Additional file 1: **Figures S1**–**S6.** and **Tables S1.** and **S2.** This file contains the supplementary/additional figures and tables. (DOCX 1940 kb)

## Data Availability

Genomics datasets have been submitted to EBI ENU with project ID PRJEB28732.
