# Supplementary material for: Skin microbiome modulation induced by probiotic solutions
Source: Microbiome. 2019 Jun 24;7:95. doi: 10.1186/s40168-019-0709-3 (PMC6591853; doi:10.1186/s40168-019-0709-3)
Supplement: Supplementary file 1 [file 40168_2019_709_MOESM1_ESM.docx]

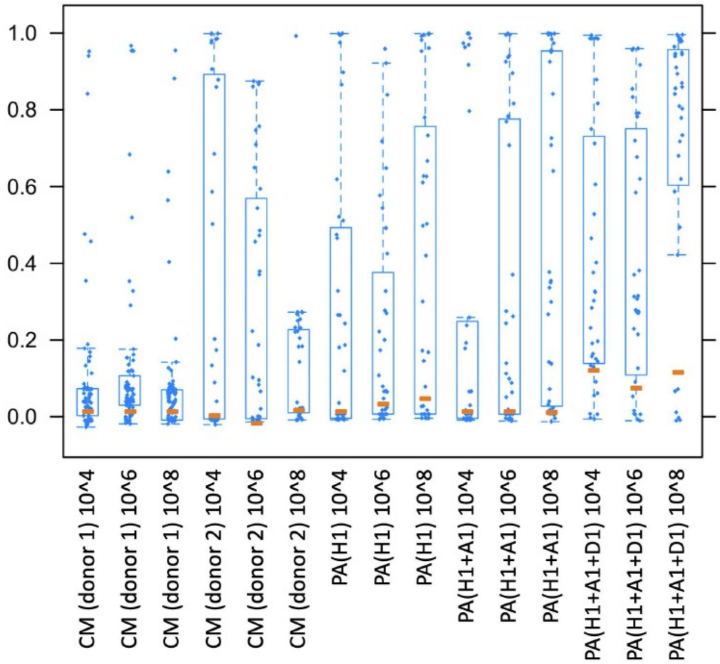

Figure S1. Engraftment of the different probiotic solutions used. Different types and concentrations. In orange, see indicated levels for engraftment in negative control areas.

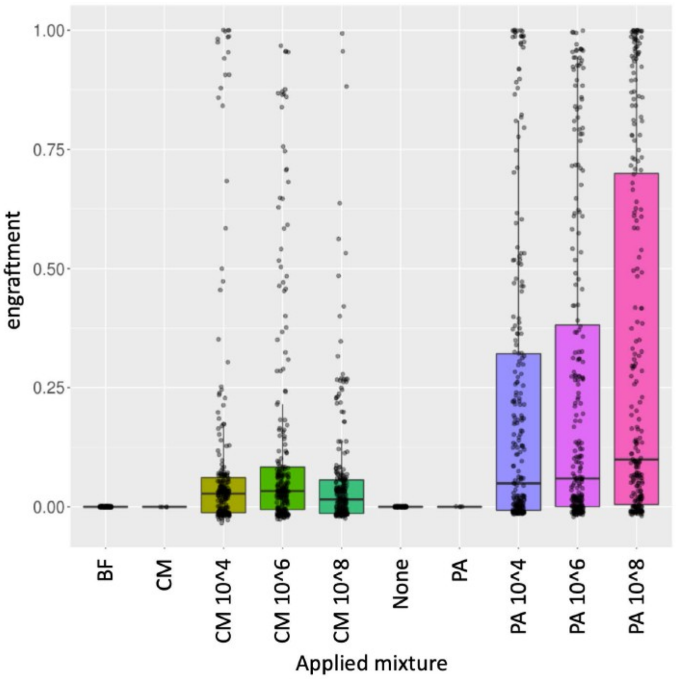

Figure S2. Engraftment of the different probiotic solutions used grouped by concentrations.

Figure S3. *C. acnes* substrain composition presented by the donor solutions. Relative counts are presented in the heatmap (#reads specific for substrain SLST tag/#average number of reads for any SLST tag).

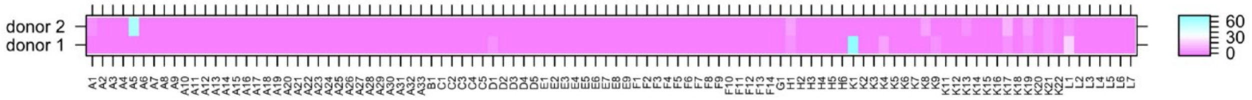

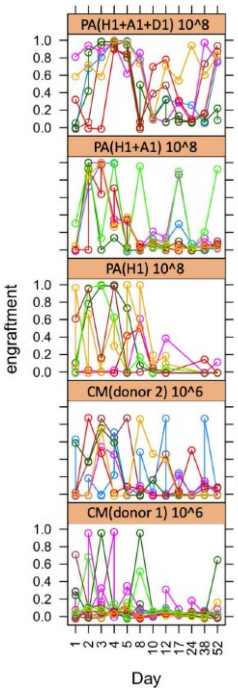

Figure S4. Engraftment level of different probiotic solutions at different days of application. Different colors in each panel represent individual subjects.

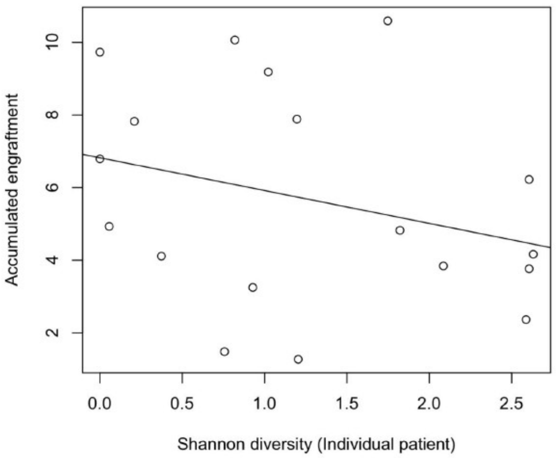

Figure S5. Engraftment level for different Shannon diversity index. We do not see a significant association between accumulated engraftment for patients and diversity of their microbiome.

```
Adjusted R-squared:  0.02805
```

A. Distribution of relative abundances for the 10 most common genera.

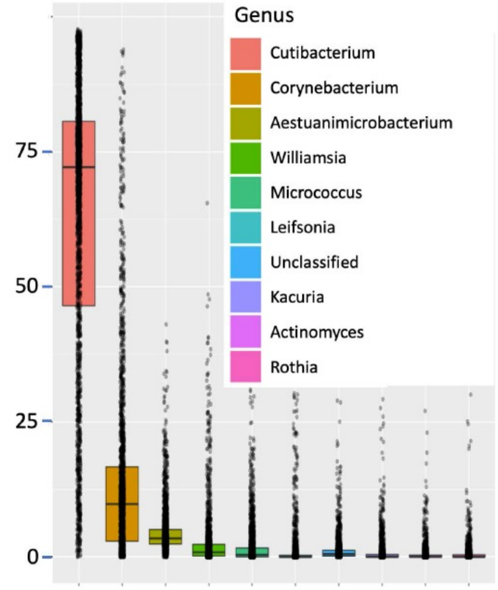

B. Total normalized abundances per sample for 3 OTUs

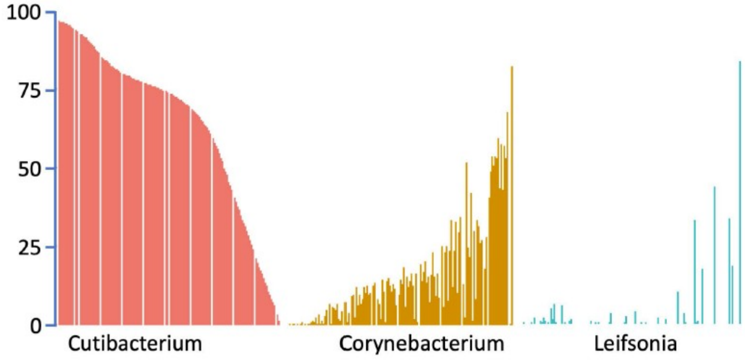

Figure S6. Relative abundances observed in 16S. A) Average relative abundance for the 10 most common genera. Values above bars are the percentage of samples in which the given genus appears. B) Relative abundance per sample, ordered by value for most common genus, Cutibacterium. Leifsonia is correlated with presence of Corynebacterium and anti-correlated with Cutibacterium

Probiotic solution name	Sected C. acnes strains	concentration/CFU.mL^{-1}
PA(H1+A1+D1) 10^8	Mix of strains H1, A1 and D1	1.00E+08
PA(H1+A1) 10^8	Mix of strains H1 and A1	1.00E+08
PA(H1) 10^8	Strain H1	1.00E+08
PA(H1+A1+D1) 10^6	Mix of strains H1, A1 and D1	1.00E+06
PA(H1+A1) 10^6	Mix of strains H1 and A1	1.00E+06
PA(H1) 10^6	Strain H1	1.00E+06
PA(H1+A1+D1) 10^4	Mix of strains H1, A1 and D1	1.00E+04
PA(H1+A1) 10^4	Mix of strains H1 and A1	1.00E+04
PA(H1) 10^4	Strain H1	1.00E+04
CM (donor 1) 10^8	Complete microbiome from donor 1	1.00E+08
CM (donor 1) 10^6	Complete microbiome from donor 1	1.00E+06
CM (donor 1) 10^4	Complete microbiome from donor 1	1.00E+04
CM (donor 2) 10^8	Complete microbiome from donor 2	1.00E+08
CM (donor 2) 10^6	Complete microbiome from donor 2	1.00E+06
CM (donor 2) 10^4	Complete microbiome from donor 2	1.00E+04

Table S1. List of probiotic solutions used in the study. PA mixtures are composed of H1 as main component and amounts of D1 (similar as H1) and A1 (~1%).

SLST type	ribotype
A1	type IA
A2	type IA
A28	*type IA*
A32	*type IA*
A5	*type IA*
C1	type IA
C3	*type IA*
D1	type IA
E3	type IA
F4	type IA
H1	type IB
K1	type II
K7	*type II*
K8	type II
L1	type III

Table S2. List of equivalent ribotype for each SLST type described in the manuscript. Ribotype equivalences were collected from Scholz et al, Plos ONE 2014
Equivalences in italics were inferred from SLST phylogeny supplied by the authors of the manuscript on the last version (23rd January 2019) of the SLST prediction server.
